# Blood Plasma Biomarkers for Woody Breast Disease in Commercial Broilers

**DOI:** 10.3389/fphys.2021.712694

**Published:** 2021-07-22

**Authors:** Byungwhi Kong, Bhuwan Khatri, Seong Kang, Stephanie Shouse, Hakeem Kadhim, Michael Kidd, Kentu Lassiter, Joseph Hiltz, Barbara Mallmann, Sara Orlowski, Nicholas Anthony, Walter Bottje, Wayne Kuenzel, Casey Owens

**Affiliations:** ^1^Department of Poultry Science, Center of Excellence for Poultry Science, University of Arkansas, Fayetteville, AR, United States; ^2^Veterinary Medicine College, University of Thi-Qar, Nasiriyah, Iraq

**Keywords:** woody breast, blood plasma, biomarker, proteomics, metabolomics

## Abstract

Woody breast (WB) myopathy results in poor muscle quality. The increasing incidence of WB over the last several years indicates a need for improved prediction or early diagnosis. We hypothesized that the use of body fluids, including blood, may be more suitable than breast muscle tissue in developing a minimally invasive diagnostic tool for WB detection. To identify potential early-age-biomarkers that may represent the potential onset of WB, blood samples were collected from 100, 4 wks old commercial male broilers. At 8 wks of age, WB conditions were scored by manual palpation. A total of 32 blood plasma samples (eight for each group of WB and non-WB control birds at two time points, 4 wks and 8 wks) were subjected to shotgun proteomics and untargeted metabolomics to identify differentially abundant plasma proteins and metabolites in WB broilers compared to non-WB control (Con) broilers. From the proteomics assay, 25 and 16 plasma proteins were differentially abundant (*p* < 0.05) in the 4 and 8 wks old samples, respectively, in WB compared with Con broilers. Of those, FRA10A associated CGG repeat 1 (FRAG10AC1) showed >2-fold higher abundance in WB compared with controls. In the 8 wks old broilers, 4 and 12 plasma proteins displayed higher and lower abundances, respectively, in WB compared with controls. Myosin heavy chain 9 (MYH9) and lipopolysaccharide binding protein (LBP) showed more than 2-fold higher abundances in WB compared with controls, while transferrin (TF) and complement C1s (C1S) showed more than 2-fold lower abundances compared with controls. From the untargeted metabolomics assay, 33 and 19 plasma metabolites were differentially abundant in birds at 4 and 8 wks of age, respectively, in WB compared with controls. In 4 wks old broilers, plasma 3-hydroxybutyric acid (3-HB) and raffinose concentrations showed the highest and lowest fold changes, respectively, in WB compared with controls. The blood plasma 3-HB and raffinose concentrations were confirmed with targeted biochemical assays. Blood biomarkers, such as 3-HB and raffinose, may be suitable candidate targets in the prediction of WB onset at early ages.

## Introduction

The incidence of woody breast (WB) myopathy has increased in the last several years, possibly in response to genetic selection for growth performance and a shift to heavier market weight birds (Velleman, 2015; Kuttappan et al., 2017; Lake and Abasht, 2020). Extensive investigations on WB myopathy characterized etiopathogenic mechanisms in breast muscle during the disease progression (Velleman and Clark, 2015; Abasht et al., 2016; Papah et al., 2017; Sihvo et al., 2017; Brothers et al., 2019; Greene et al., 2019; Maharjan et al., 2019; Petracci et al., 2019; Lake and Abasht, 2020). Those muscular mechanisms include phlebitis and inflammation, oxidative stress and metabolic dysfunction, myofiber degeneration, lipid deposition and development of hard pectoral muscle, resulting in poor muscle quality. The exact cause of WB myopathy is still unclear and most genetic, nutritional, and physiological studies have been conducted with breast muscle tissues. For early diagnosis or prediction for later onset of WB myopathy, less invasive body fluids including blood may become more suitable than breast muscle tissues in developing a diagnostic tool. A recent study using targeted metabolomics reported differentially abundant blood metabolites including homocysteine, cyclic GMP, trimethylamine N-oxide, tyramine, carnitine, and acetylcarnitine, which are mainly connected to the cardiovascular system (Maharjan et al., 2019). Less invasive biomarkers contained in blood samples, such as plasma proteins and metabolites, can be effective for pre-diagnosis of WB during the normal growth phase of broilers. The purpose of this study was to identify differentially abundant blood plasma proteins and metabolite biomarkers in efforts to explore identifying early-age-biomarkers that can represent the potential onset of WB using shotgun proteomics and untargeted metabolomics methods, respectively.

## Materials and Methods

### Ethics Statement

The care and experimental use of animal protocols were accepted by the University of Arkansas Institutional Animal Care and Use Committee. Animals were maintained according to a standard management program at the Poultry Farm, University of Arkansas.

### Animals and Sample Preparation

Commercial broiler chickens were raised in an environmentally controlled room. A standard commercial diet was fed *ad libitum*. At 4 and 8 wks old, blood from male birds was collected consistently and gently by the same person from the wing vein (brachial vein) to minimize human presence and handling stress. At 8 wks of age, live birds were clinically inspected by physical palpation and visual examination of the breast muscle (pectoralis major). The correlations between WB palpation and filet WB scores are known to be increased with increasing bird age (Mallmann, 2019), indicating that the physical palpation at 8 wks of age is suitable to determine WB scores. The palpation scores for WB ranged from 0 (no WB) to 3 (severe WB), with a score of 1 being mild WB and a score of two being moderate WB (Mallmann, 2019; Kang et al., 2020). Post palpation, the samples were grouped into control (Con, *n* = 8; WB score = 0) and WB (*n* = 8; WB score ≥ 2) birds. Blood samples were centrifuged and plasma was stored at −20°C until examined for metabolomics and proteomics.

### Shotgun Proteomics

Plasma collected from Con and WB broilers at 4 and 8 wks of age (32 plasma samples in total) were subjected to shotgun proteomics analysis by trypsin digestion and tandem mass spectrometry (MS/MS) at the University of Arkansas Medical Science (UAMS) Proteomics Core Facility (Little Rock, AR). Raw mass spectrometric data were analyzed by database searching using the Mascot (Matrix Science, Boston, MA) search engine and the UniProtKB (http://www.uniprot.org/help/uniprotkb) database. Search results were compiled using Scaffold program (Proteome Software, Portland, OR). The mass spectrometry proteomics data have been deposited to the ProteomeXchange Consortium via the PRIDE (Perez-Riverol et al., 2019) partner repository with the dataset identifier PXD026896 and 10.6019/PXD026896. Normalization and statistical analyses are described in the “Statistical Analyses” section.

### Untargeted Metabolomics

Plasma samples used for proteomics analysis were also subjected to primary metabolism analysis by GC-TOF MS at the UC Davis West Coast Metabolomics Center (Davis, CA). Normalization and statistical analyses are described below in the “Statistical Analyses” section.

### Plasma Raffinose Assay

Plasma raffinose concentration was determined using raffinose/D-galactose assay kit (Megazyme, Ireland) following manufacturer's instructions with modifications for use in a microplate. Briefly, 20 μl plasma samples were mixed with either 10 μl α-galactosidase (pH 4.6) or 10 μl distilled water for determining concentration of raffinose + free D-galactose or free D-galactose only, respectively, and mixtures were incubated at 25°C for 20 min. Distilled water was used as blank control. Next, 20 μl buffer containing sodium azide, 200 μl distilled water, and 10 μl NAD^+^ solution, were added, mixed, and absorbances read at 340 nm (A1 read) after 3 min. Then, 2 μl D-galactose dehydrogenase plus galactose mutarotase suspension was added, mixed, incubated at 40°C for 20 min, and absorbances read at 340 nm at the end of the reaction (A2 read). Raffinose concentration was calculated by:

$${\text{C}}_{\text{raffinose}}(\text{g}/\text{L})=(\text{V}\times \text{MW}\times \Delta {\text{A}}_{\text{raffinose}})\text{/}(\varepsilon \times \text{d}\times \;\text{v})$$

V = final volume [ml]

MW = molecular weight of the substance (504.5 for raffinose) assayed [g/mol]

$$\begin{array}{l} \Delta {\text{A}}_{\text{raffinose}}=\Delta {\text{A}}_{\text{raffinose}+\text{freeD}-\text{galactose}}-\Delta {\text{A}}_{\text{free}\;\text{D}-\text{galactose}}\\ \;\Delta \text{A}=\text{A}2-\text{A}1 \end{array}$$

ε = extinction coefficient of NADH at 340 nm = 6300 [l × mol^−1^ × cm^−1^]

d = light path [cm] = 1

v = sample volume [ml] = 0.02

The 96 well plate was read twice with a Synergy HT multimode microplate reader (BioTek, Winooski, VT). Average concentration of individual samples was used with standard error of mean (SE).

### Plasma 3-Hydroxybutyric Acid Assay

Plasma 3-hydroxybutyric acid (3-HB) concentration was determined using D-3-hydroxybutyric acid assay kit (Megazyme, Ireland) following manufacturer's instructions for use in a microplate. Briefly, 10 μl plasma samples were mixed with 200 μl distilled water, 50 μl buffer mix containing sodium azide, 20 μl NAD^+^ plus iodonitrotetrazolium chloride, and 2 μl diaphorase suspension, mixed, and absorbances read at 492 nm after 3 min (A1 read). Distilled water (210 μl), and 10 μl standard solution (0.06 mg/ml; provided by manufacturer) were used as blank control and internal standard concentration, respectively. Next, 2 μl 3-Hydroxybutyrate dehydrogenase suspension (3-HBDH) was added, mixed, and incubated at 25°C for ~6 min, and absorbances read at 492 nm at the end of the reaction (A2 read). D-3-hygroxybutyric acid concentration was calculated by:

$$\begin{array}{l} {\text{C}}_{\text{D}-3-\text{hydroxybutyricacid}}(\text{g}/\text{L})\\ \;=(\Delta {\text{A}}_{\text{sample}}\times \text{g}/\text{L}\;\text{standard})/\Delta {\text{A}}_{\text{standard}}\\ \;\Delta A=\text{A2}-\text{A1} \end{array}$$

The 96 well plate was read twice with a Synergy HT multimode microplate reader (BioTek, Winooski, VT). Average concentration of individual samples was used with SE.

### Statistical Analyses

Raw spectral counts of either proteomics or metabolomics were transformed to log_2_ values and normalized by Loess method using JMP Genomics (SAS Institute, Cary, NC). Differential abundances between WB and Con were calculated using log_2_fold change (FC), indicating |log_2_FC of 1.0| = |FC of 2.0| in numeric value. The Student's *t*-test was used separately for proteomics or metabolomics in comparing Con and WB samples. Proteins or metabolites showing *p* < 0.05 in the comparison between Con and WB were considered differential abundance. The *p*-value correction (FDR calculation) by multiple tests was not applied in this study since we used a less stringent approach on a molecule by molecule basis, and this allowed us to import more informative lists. For biochemical assays for raffinose and 3-HB, the Student's *t*-test comparing Con and WB was used and *p* < 0.05 was considered as significant differences.

## Results and Discussion

### Body Weight

Body weights (average ± standard deviation; SD) at 8 wks for Con and WB broilers were 3334 ± 231 g and 3462 ± 261 g, respectively, indicating WB group broilers were slightly heavier (3.8%) than non-WB controls (*p* > 0.05).

### Plasma Protein Biomarkers

Plasma samples (8 broilers × 2 groups × 2 time points) collected from commercial broilers, 4 and 8 wks of age, were subjected to both shotgun proteomics and primary metabolomics. From the proteomics assay, 25 and 16 plasma proteins were differentially abundant (*p* < 0.05) at 4 (Table 1) and 8 wks of age (Table 2), respectively, in WB compared with controls. In 4 wks old broilers, 11 and 14 plasma proteins showed higher and lower abundances, respectively, in WB compared with controls. Assuming greater differences in abundance of markers, their greater effects on physiology as well as improvements in their diagnostic method, has resulted in markers showing greater differences between WB and controls. Therefore, the value of log_2_FC >1.0 were considered first in this analysis. Of differentially abundant blood proteins, FRA10AC1 (log_2_FC = 1.2) showed log_2_FC higher than 1.0 abundance in WB broilers compared with controls, while none of the proteins showed log_2_FC < -1.0 abundance compared with controls. In 8 wk old broilers, 4 and 12 plasma proteins in WB showed higher and lower abundance, respectively, compared with controls. MYH9 (log_2_FC = 2.9) and LBP (log_2_FC = 1.4) showed log_2_FC higher than 1.0 abundance in WB broilers compared with controls, while TF (log_2_FC = −1.9) and C1S (log_2_FC = −1.0) showed log_2_FC <-1.0 abundance compared with controls. Interestingly, COL1A1, COL1A2, and CCL26 in plasma samples in both 4 and 8 wk old WB broilers were lower compared to controls, indicating consistently lower concentrations in WB plasma. However, the fold change values were marginal (−1 < log_2_FC <1.0) for all three plasma proteins.

**Table 1:** Plasma protein biomarkers in WB at 4 wks of age compared to Con.

**Symbol**	**Entrez gene name**	**Log_**2**_FC**	***P*-value**
FRA10AC1	FRA10A associated CGG repeat 1	1.2	0.0402
GAL9	Gallinacin 9	0.9	0.0456
TALDO1	Transaldolase 1	0.6	0.0022
APOA5	Apolipoprotein A5	0.5	0.0163
ACAT2	Acetyl-CoA acetyltransferase 2	0.5	0.0008
CR1L	Complement C3b/C4b Receptor 1 Like	0.4	0.0246
CKB	Creatine kinase B	0.3	0.0486
FETUB	Fetuin B	0.2	0.0392
LDHB	Lactate dehydrogenase B	0.2	0.0145
GOT1	Glutamic-oxaloacetic transaminase 1	0.2	0.0363
HSPA5	Heat shock protein family A (Hsp70) member 5	0.1	0.0215
GLB1L	Galactosidase beta 1 like	−0.2	0.0160
TIMP2	TIMP metallopeptidase inhibitor 2	−0.2	0.0244
COL5A1	Collagen type V alpha 1 chain	−0.2	0.0451
SERPINF1	Serpin family F member 1	−0.2	0.0153
DKK3	Dickkopf WNT signaling pathway inhibitor 3	−0.3	0.0466
CHL1	Cell adhesion molecule L1 like	−0.4	0.0383
SERPINA1	Serpin family A member 1	−0.4	0.0466
MMP9	Matrix metallopeptidase 9	−0.4	0.0452
TXNDC5	Thioredoxin domain containing 5	−0.5	0.0184
COL1A2	Collagen type I alpha 2 chain	−0.5	0.0373
CCL26	Chemokine ligand 26	−0.6	0.0325
EPYC	Epiphycan	−0.6	0.0214
COL1A1	Collagen type 1 alpha 1 chain	−0.7	0.0259
CRTAC1	Cartilage acidic protein 1	−0.7	0.0183

**Table 2:** Plasma protein biomarkers in WB at 8 wks of age compared to Con.

**Symbol**	**Entrez gene name**	**Log_**2**_FC**	***P*-value**
MYH9	Myosin heavy chain 9	2.9	0.0340
LBP	Lipopolysaccharide binding protein	1.4	0.0077
TKT	Transketolase	0.9	0.0018
F13A1	Coagulation factor XIII A chain	0.6	0.0458
MINPP1	Multiple inositol-polyphosphate phosphatase 1	−0.4	0.0359
CCL26	C-C motif chemokine ligand 26	−0.4	0.0491
SPP2	Secreted phosphoprotein 2	−0.5	0.0293
CDH11	Cadherin 11	−0.5	0.0393
COL1A1	Collagen type I alpha 1 chain	−0.6	0.0118
PPIC	Peptidylprolyl isomerase C	−0.6	0.0120
COL1A2	Collagen type I alpha 2 chain	−0.6	0.0165
GC	GC, vitamin D binding protein	−0.7	0.0202
A2M	Alpha-2-macroglobulin	−0.7	0.0417
CDH5	Cadherin 5	−0.8	0.0060
C1S	Complement C1s	−1.0	0.0396
TF	Transferrin	−1.9	0.0365

FRA10A associated CGG repeat 1 (FRA10AC1; log_2_FC = 1.2 higher in WB broilers), which is ubiquitously expressed and mainly localized in nucleoplasm (Sarafidou et al., 2004), has not been determined in blood plasma. Instead, circulating FRA10AC1 mRNA was identified as one of the RNA biomarkers for melanoma (Sole et al., 2019), suggesting that FRA10AC1 mRNA and protein can be circulating biomarkers for WB myopathy, though their functional role in blood is unknown. A subtype (COL1A1), of collagens which showed marginal differences in WB plasma in both 4 and 8 wks of age, was reported as a potential biomarker for heart failure progression (Hua et al., 2020). Likewise, C-C motif chemokine ligand 26 (CCL26) in blood is known to be significantly correlated with the disease activity of atopic dermatitis (Kagami et al., 2003). To use these blood biomarkers for early diagnostic purpose, highly specific (for FRA10AC1 as chicken specific antibody is not available), and sensitive assay methods need to be developed to discern the marginal differences in blood plasma.

Higher differential abundances of MYH (log_2_FC = 2.9), LBP (log_2_FC = 1.4), TF (log_2_FC = −1.9), and C1S (log_2_FC = −1.0) were detected in WB broilers in the late growth phase only (8 wks of age), thus these markers may not be suitable for early diagnosis for WB. Regarding plasma myosin species, it has been reported that red blood cells (RBCs) contain non-muscle myosins (Hammer, 2018). The function of non-muscle myosins in RBCs is different from its original function in normal cells reflecting the RBC's unusual actin organization. Thus, the inclusion of myosin subtype in blood plasma may be derived from breakdown of RBCs. Circulating lipoprotein binding protein (LBP) concentration is known to be significantly increased with type 2 diabetes and dramatically increased with obesity (Moreno-Navarrete et al., 2012). Lake et al. (2019) reported the relationship between dysregulation of lipid metabolism in WB muscle and human diabetes and further discussion will be followed elsewhere. Transferrin (TF) showed relatively large difference (log_2_FC = −1.9) in WB broilers compared with controls, indicating the late phase of WB broilers may undergo hypoxic conditions, which were previously reported as a possible cause of WB incidence (Sihvo et al., 2018). Complement C1s (C1S) is a protease enzyme that functions in immunological host defense against microbial infection (Lu and Kishore, 2017), reflecting increased inflammatory responses in WB broilers in the late growth phase.

### Plasma Metabolite Biomarkers

Untargeted metabolomics assay for primary metabolism analysis was conducted to identify differentially abundant plasma metabolites in WB broilers compared with controls. The same plasma samples used in the proteomics assays were used for metabolomics analysis. Overall, 33 and 19 plasma metabolites were differentially abundant (*p* < 0.05) at 4 (Table 3) and 8 wks old (Supplementary Material), respectively, in WB broilers compared with controls. In 4 wks old broilers, 4 and 10 metabolites showed log_2_FC higher than 1.0 and lower than −1.0 abundances, respectively, in WB broilers compared with controls. In 8 wks old broilers, plasma metabolites displayed lowered differential abundance in WB compared with controls (Supplementary Material). Table 3 shows log_2_FC more than 1.0 or <-1.0 differentially abundant metabolites in 4 wks old WB broilers in addition to the corresponding log_2_FC and *p*-values in plasma samples taken at 8 wks of age. Plasma raffinose contents showed the lowest FC values in WB broilers compared with controls at 4 wks of age. Furthermore, raffinose contents were significantly lower in plasma samples at 8 wks of age. Results suggest that raffinose may be an effective plasma biomarker to diagnose WB in broilers during their early growth phase.

**Table 3:** Plasma metabolite biomarkers in WB at 4- and 8 wks of age compared to Con.

**Metabolite name**	**4 wks**		**8 wks**	
	**Log_**2**_FC**	***P*-value**	**Log_**2**_FC**	***P*-value**
3-hydroxybutyric acid	1.6	0.0152	0.7	0.1042
Lysine	1.4	0.0263	−0.3	0.6155
Uridine	1.3	0.0228	0.2	0.4926
2-aminobutyric acid	1.0	0.0362	0.4	0.0949
Mucic acid	−1.1	0.0369	−0.6	0.0319
Asparagine	−1.1	0.0364	−0.1	0.7950
Orotic acid	−1.2	0.007	−0.7	0.0819
Pentitol	−1.2	0.0356	−0.4	0.0655
Xanthine	−1.3	0.0105	−0.3	0.4404
Xylonic acid	−1.3	0.0273	−0.5	0.0258
Butane-2,3-diol	−1.4	0.0406	0.4	0.3412
Pinitol	−1.6	0.0450	−0.5	0.0328
Beta-gentiobiose	−2.7	0.0345	−0.8	0.0326
Raffinose	−3.3	0.0441	−1.1	0.0491

### Plasma Concentration for Raffinose and 3-Hydroxybutyric Acid

**Figure 1:**
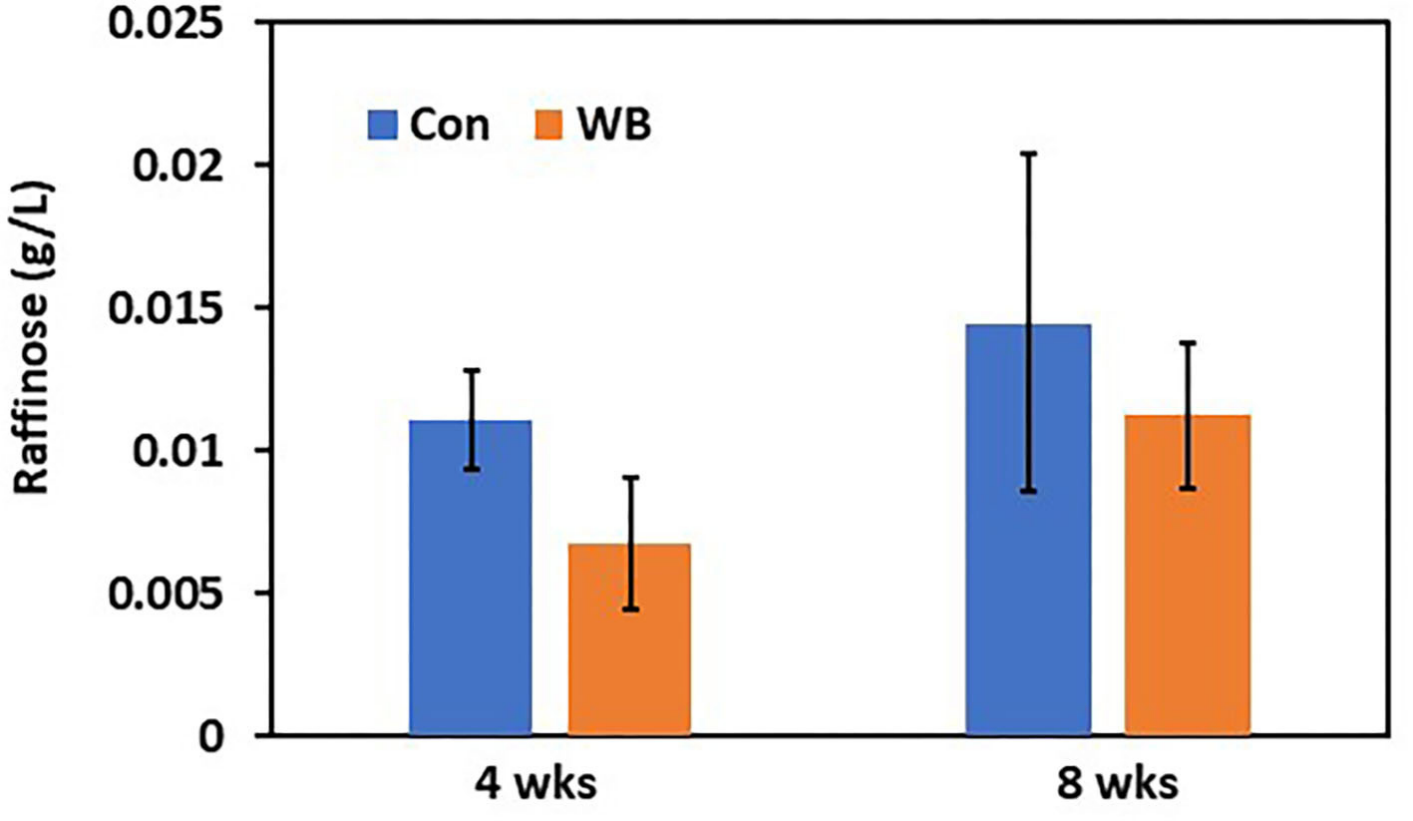
Plasma raffinose concentration in WB and controls. Raffinose concentrations were determined at 4- and 8 wks of age. Bars indicate mean ± SE.

**Figure 2:**
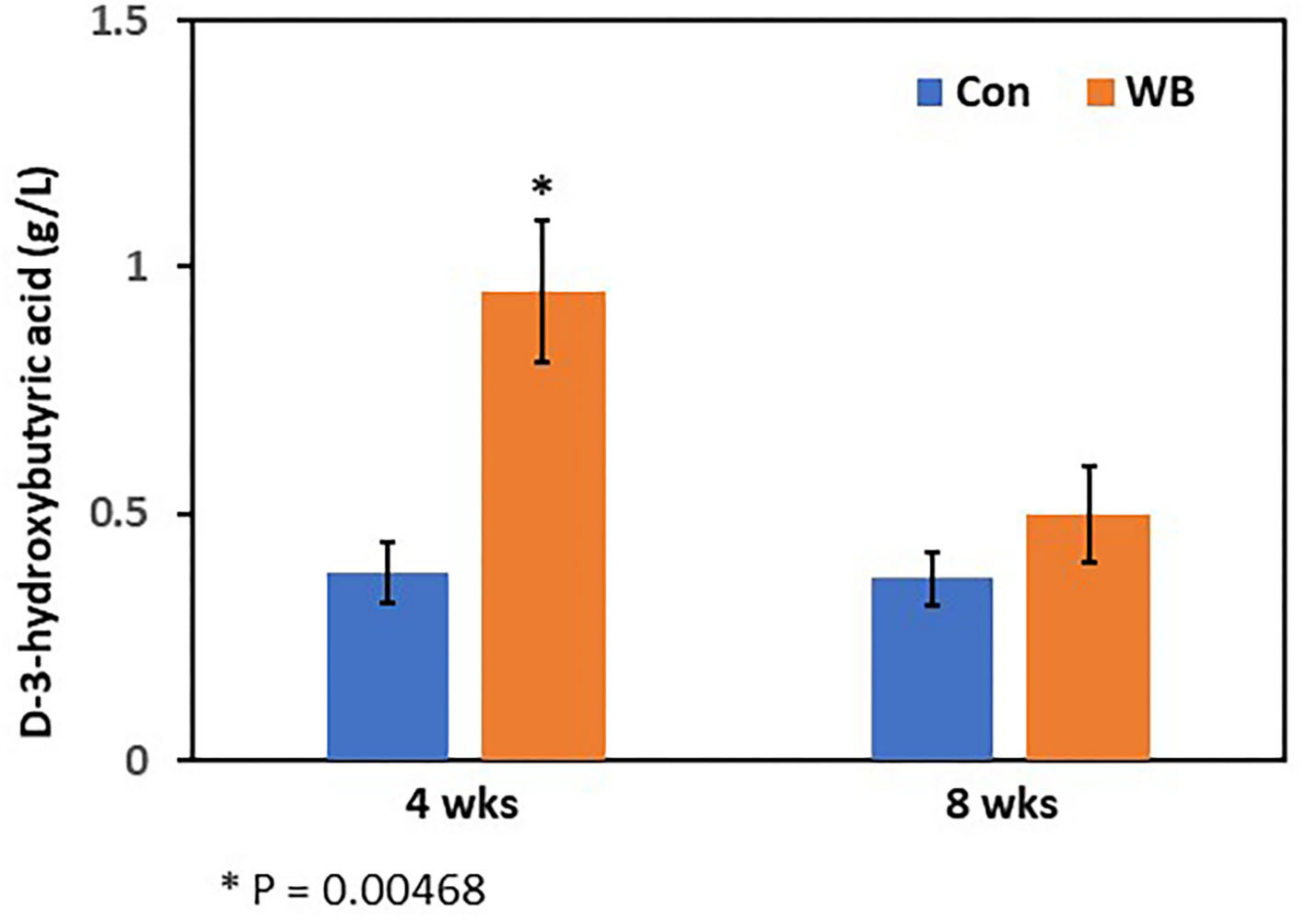
Plasma 3-hydroxybutyric acid concentration in WB and controls. The 3-HB concentrations were determined at 4- and 8 wks of age. Bars indicate mean ± SE.

To verify differential abundance of plasma raffinose as the lowest FC metabolite and 3-HB as the highest FC metabolite in WB broilers, concentrations for raffinose and 3-HB were determined using raffinose/D-galactose assay (Figure 1) and D-3-hydroxybutyric acid assay methods (Figure 2), respectively. Raffinose plasma concentrations in WB broilers at 4 wks of age was lower (0.006 ± 0.0023 g/L) than those of controls (0.01 ± 0.0017 g/L) consistent with the result of differential abundance in the metabolomics assay. At 8 wks of age, plasma raffinose concentrations in both Con and WB were higher than 4 wk old samples and those in the WB group were slightly lower than controls (p > 0.05) consistent with the metabolomics data (Figure 1). However, the concentration of plasma raffinose showed very low levels (0.006–0.012 g/L), suggesting that a more sensitive measuring tool is needed to detect plasma raffinose.

Concentrations of plasma 3-HB in WB broilers at 4 wks of age (0.95 ± 0.15 g/L) were higher (*p* = 0.005) than those of controls (0.38 ± 0.06 g/L), which is also consistent with the result of the metabolomics analysis (Figure 2). In 8 wk old broilers, plasma 3-HB concentrations were slightly higher than in the Con group (*p* > 0.05) consistent with the metabolomics data.

Raffinose, a trisaccharide composed of galactose, glucose, and fructose, is mainly derived from soybean meal and is not digested in chicken gut due to lack of enzymes digesting raffinose (Valentine et al., 2017). The presence of plasma raffinose implicated two possible causes including leaky gut syndrome and/or differential microbial communities. Leaky gut syndrome, which is frequently observed in broiler production, may be associated with the incidence of WB. Differential microbial communities with variable abilities to digest raffinose may influence the incidence of WB. In fact, an earlier report (Zhang et al., 2019) characterized differential proportions of microbial species within the microbiome in WB compared with non-WB broilers, showing that *Selenomonas bovis* and *Bacteroids plebeius* were higher in caeca of WB broilers. Interestingly, *S. bovis* can breakdown raffinose (Zhang and Dong, 2009). Collectively, WB broilers may digest more raffinose by *S. bovis* in the gut, resulting in a higher incidence of WB.

The 3-hydroxybutyric acid (3-HB, also known as β-hydroxybutyric acid), together with acetone, and acetoacetic acid, is one of the ketone bodies that are a group of organic compounds of intermediary fat metabolism. High concentrations of ketone bodies decrease the rate of β-oxidation of fatty acids (Cadorniga-Valino et al., 1997). In human, circulating 3-HB levels > 3 mmol/L in children or >3.8 mmol/L in adults are considered the clinically significant level in diabetic ketoacidosis (DKA), whereas below 0.5 mmol/L is considered as normal. With starvation, levels can rise up to 6 mmol/L (Depczynski et al., 2019). Dogs with a blood ketone concentration >3.5 mmol/L are at higher risk for developing DKA (Di Tommaso et al., 2009). In this study, when examined at 4 wks of age, WB broilers showed 0.95 g/L (~9.12 mmol/L in average; MW of 3-HB = 104.1) while non-disease controls showed 0.38 g/L (~3.65 mmol/L in average). This is a striking result as higher 3-HB concentrations (~9.12 mmol/L) at this early age of WB broilers is comparable to a severe clinical condition of DKA in humans (>3.8 mmol/L) and dogs (3.5 mmol/L). Moreover, non-WB broilers still showed the sub-clinical level of circulating 3-HB concentration (~3.65 mmol/L). This may be correlated with the rapid growth in early life and altered muscular structures including decreased capillary blood supply, reduced connective tissue spacing between myofibers and muscle fiber bundles, and increased degeneration of myofibers, that occurred from intense growth-related selection resulting in modern broilers becoming more susceptible to the incidence of WB myopathy. Elevated levels of basal 3-HB in blood may be evidence of higher susceptibility of WB in modern broilers.

3-hydroxybutyric acid inhibits lipolysis via the PUMA-G receptor (Kimura et al., 2011) and reduces total energy expenditure by inhibiting short-chain fatty acid signaling through GPR41 (Taggart et al., 2005). A higher concentration of 3-HB in the blood of cows is considered an indicator of subclinical ketosis (Puppel and Kuczynska, 2016), and thus can be used as a biomarker for early diagnosis of ketosis (Puppel et al., 2019). In chicken, 3-HB concentration in blood after hatching is dramatically decreased to <20% of the concentration at 3 days and maintained at a steady level (Ohtsu et al., 2003). Therefore, higher concentrations of 3-HB in WB chicken plasma may indicate high ketoacidosis, suggesting that dysregulation of fat metabolism is a potential cause of WB. Increased concentration of 3-HB is a biomarker of increased ketosis showing subclinical or clinical conditions of metabolic diseases, such as diabetes in human (Depczynski et al., 2019). Lake et al. (2019) suggested that the altered physiological pathways, including dysregulation of lipid metabolism followed by lipid accumulation identified as early as 2 wks of age found in WB muscle were similar to those found in human diabetes (Lake and Abasht, 2020). Additional evidence of altered lipid metabolism in WB muscle associated with feed efficiency was revealed as metabolic signatures of susceptibility to muscle disorders in modern broilers (Abasht et al., 2019). Moreover, this result supports a previous report showing higher 3-HB concentration in WB muscle compared to non-WB muscle (Abasht et al., 2016). Taken together, these observations of higher concentrations of blood 3-HB in WB broilers suggests it is a potential less-invasive biomarker for early diagnosis of WB incidence.

## Conclusion

Woody breast is a myopathy that exhibits greatest severity in the late growth phase of broilers with an etiology that may commence early in development (Lake and Abasht, 2020). Early diagnosis, when cases are mild, can be difficult to detect via palpation and thus, plasma biomarkers could be used to develop a less invasive diagnostic tool for early detection of WB, as reported in this study. Blood biomarkers, such as 3-HB and raffinose, are candidate targets to predict WB susceptibility. Further research evaluating a larger number of broilers and different broiler populations is needed to validate the candidate biomarkers. In this study, only two metabolites were confirmed by biochemical assays and other differentially abundant proteins (e.g., FRA10AC1) and metabolites will be examined with targeted biochemical assays in future studies.

## Data Availability Statement

The proteomics datasets presented in this study can be found in online repositories. The names of the repository/repositories and accession number(s) can be found at: ProteomeXchange with identifier PXD026896 (Project DOI 10.6019/PXD026896).

## Ethics Statement

The animal study was reviewed and approved by University of Arkansas Institutional Animal Care and Use Committee.

## Author Contributions

BKo, BKh, SK, WB, WK, and CO designed the experiments. BKo, BKh, SK, SS, HK, MK, KL, SO, JH, NA, WB, and WK performed the chicken experiments and analyzed the data. BM and CO conducted chicken processing and woody breast scoring. BKo wrote the draft of this manuscript. All authors have read, edited, and approved the final manuscript.

## Conflict of Interest

The authors declare that the research was conducted in the absence of any commercial or financial relationships that could be construed as a potential conflict of interest.
